# Anticonvulsant and Antioxidant Effects of *Tilia americana* var. *mexicana* and Flavonoids Constituents in the Pentylenetetrazole-Induced Seizures

**DOI:** 10.1155/2014/329172

**Published:** 2014-08-13

**Authors:** Noemí Cárdenas-Rodríguez, María Eva González-Trujano, Eva Aguirre-Hernández, Matilde Ruíz-García, Aristides Sampieri, Elvia Coballase-Urrutia, Liliana Carmona-Aparicio

**Affiliations:** ^1^Laboratory of Neurochemistry, National Institute of Pediatrics, 04530 Mexico City, DF, Mexico; ^2^Laboratory of Neuropharmacology of Natural Products, National Institute of Psychiatry, 14370 Mexico City, DF, Mexico; ^3^Ecology and Natural Resources Department, Faculty of Sciences, National Autonomous University of Mexico, 04150 Mexico City, DF, Mexico; ^4^Neurology Service, National Institute of Pediatrics, 04530 Mexico City, DF, Mexico; ^5^Laboratory of Molecular Biology and Genomic, Faculty of Sciences, National Autonomous University of Mexico, 04150 Mexico City, DF, Mexico

## Abstract

*Tilia* genus is commonly used around the world for its central nervous system properties; it is prepared as tea and used as tranquilizing, anticonvulsant, and analgesic. In this study, anticonvulsant activity of the *Tilia americana* var. *mexicana* inflorescences and leaves was investigated by evaluating organic and aqueous extracts (100, 300, and 600 mg/kg, i.p.) and some flavonoids in the pentylenetetrazole-induced seizures in mice. Moreover, antioxidant effect of these extracts and flavonoids was examined in an *in vitro* study by using spectrophotometric technique. Significant activity was observed in the methanol extract from inflorescences. An HPLC analysis of the methanol extract from inflorescences and leaves of *Tilia* allowed demonstrating the respective presence of some partial responsible flavonoid constituents: quercetin (20.09 ± 1.20 *μ*g/mg and 3.39 ± 0.10 *μ*g/mg), rutin (3.52 ± 0.21 *μ*g/mg and 8.94 ± 0.45 *μ*g/mg), and isoquercitrin (1.74 ± 0.01 *μ*g/mg and 1.24 ± 0.13 *μ*g/mg). In addition, significant but different antioxidant properties were obtained among the flavonoids and the extracts investigated. Our results provide evidence of the anticonvulsant activity of *Tilia* reinforcing its utility for central nervous system diseases whose mechanism of action might involve partial antioxidant effects due to the presence of flavonoids.

## 1. Introduction

The* Tilia* genus belongs to the Tiliaceae family, which consists of 25–80 species, and it is mainly distributed in Europe and Asia, with a few species in North America [1, 2]. In México,* T. americana* is known as* Tilia* and it is distributed in 14 states across both the northern and southern parts of the country [3]. This plant has a wide geographical distribution; however, the populations of this species are confined to the lower mountainous forest that covers less than 1% of Mexican territory [4].

Infusions of* Tilia* are widely used in traditional medicine in Europe and Latin America for the treatment of enterocolitis, gastroenteritis and liver, and renal colic, but mainly due to its tranquilizing activity [5–9]. Phytochemical studies have demonstrated that the* Tilia* genus synthesizes hydrocarbons, esters, aliphatic acids, polyphenols, and terpenoids [10–12] with demonstrated heterogeneity in their flavonoid patterns when flowers and leaves are sampled independently [13–15]. Nevertheless, the knowledge of the potential contribution of the differences in the flavonoid pattern between inflorescences and leaves of* Tilia* on its therapeutic properties is unknown.

In previous studies, significant and dose-dependent antinociceptive and anxiolytic-like activities were demonstrated after administration of hexane, methanol, and aqueous extracts of* Tilia* inflorescences (10–300 mg/kg), where glycosides of quercetin and kaempferol were characterized as the main active compounds of the inflorescences [7, 8, 16]. Although, it has been demonstrated that these metabolites might facilitate the inhibitory response of the central nervous system (CNS), due to modulating the GABAergic and serotoninergic systems [17–19], it is unknown if these are the only possible mechanisms of action. The aim of this study was to investigate if* Tilia* organic and aqueous extracts and some of its flavonoids are able to control seizure activity in mice receiving a pentylenetetrazol (PTZ, GABA antagonist) [18, 19] and whether these extracts possess antioxidant properties.

## 2. Materials and Methods

### 2.1. Drugs

Kaempferol, quercetin, rutin, astragalin, isoquercitrin, and quercitrin standards, as well as PTZ, tween 80, and diazepam, were purchased from Sigma (Sigma-Aldrich Co., St Louis, MO, USA). Extracts were resuspended in saline solution (s.s. 0.9%, NaCl) alone or in 0.2% tween 80 in s.s. Control animals received the same volume of the vehicle via the same administration route. PTZ and flavonoids were dissolved in s.s. and diazepam in 0.2% tween 80 in s.s. Drugs were freshly prepared on the day of the experiments. All treatments were injected using an intraperitoneal (i.p.) administration within a volume of 10 mL/kg body weight.

For antioxidant analysis, reagents were used as follows: dimethyl thiourea (DMTU), nordihydroguaiaretic acid (NDGA), ascorbic acid, histidine, xylenol orange, ammonium iron (II) sulfate hexahydrate, N,N-dimethyl-4-nitrosoaniline (DMNA), catalase, xanthine, xanthine oxidase, nitroblue tetrazolium (NBT), deoxyribose, and butylated hydroxytoluene (BHT) were from Sigma Aldrich (St. Louis, MO, USA); potassium persulfate and sodium carbonate (Na_2_CO_3_) were from Mallinckrodt (Paris, KY, USA). Absolute ethanol, hydrogen peroxide (H_2_O_2_), sulfuric acid (H_2_SO_4_), methanol, ethylenediaminetetracetic acid disodium salt (EDTA), and sodium hypochlorite (NaOCl) were from JT Baker (Mexico City, Mexico). All other chemicals were reagent grade and commercially available.

### 2.2. Plant Material

Inflorescences and leaves of* Tilia americana* L. var.* mexicana* (Schltdl.) Hardin (Tiliaceae) were collected in Puebla, Mexico, in June 2010. Identification of the species was provided by Susana Valencia Avalos and a voucher specimen (number 131613) of the plant was deposited in the herbarium of the Faculty of Sciences, National Autonomous University of Mexico, Mexico City, for future reference.

### 2.3. Preparation of the Plant Extract

For the preparation of the organic extracts of* Tilia*, the air-dried powdered inflorescences (1.8 kg) were successively extracted with hexane (4 L × 3) and methanol (4 L × 3) by maceration at room temperature (22°C). Regarding the leaves, 317 g was successively extracted with hexane (1 L × 3) and methanol (1 L × 3) by maceration at room temperature (22°C). The solvents were separated from the residues by gravity filtration and then evaporated in vacuum. The final crude extracts were obtained in percentage from dry weight (% d.w.) as follows: (a) for inflorescences 25 g of a yellow greasy hexane extract (1.4% d.w.) and 130 g of a dark brown syrupy methanol extract (7.2% d.w.), (b) whereas, in the case of the leaves (317 g), the yield was hexane 3.0 g (0.95%) and 29.8 g (9.4%) methanol crude extracts.

In the case of the aqueous extracts of* Tilia* inflorescences, the air-dried powdered inflorescences (24 g) were extracted by infusion in boiling water (500 mL) for 10 min. The resulting extract was separated from its residue by gravity filtration; samples were frozen in liquid nitrogen and freeze-dried for 12 h in a Heto FD3 Lab lyophilizer. The final crude aqueous extract consisted of 1.3 g of a yellow powder or 5.3% from dry weight, whereas, in the aqueous extract from the leaves (26 g), 3.58 g of extract or 13.76% from dry weight was obtained.

### 2.4. Animals

Female Swiss albino mice (25–30 g, National Institute of Psychiatry Ramon de la Fuente Muñiz) were used for the in pharmacological tests. The animals were kept at a constant room temperature (22°C) and maintained in a 12 h/12 h light/dark cycle. All experimental procedures were carried out according to a protocol approved by the local Animal Ethics Committee (NC093280.2) in compliance with national (NOM-062-ZOO-1999) and international regulations on the care and use of laboratory animals. The animals were fed* ad libitum* with standard feed and water during the course of the study.

### 2.5. Pharmacological Evaluation

All mice were treated through a daily s.s. injection for 5 days before the treatments were administered. For each experimental procedure, animal groups consisted of six mice. Diazepam was used as a reference drug. The extracts were evaluated at doses of 100, 300, and 600 mg/kg, i.p. (these dosages were taken into account from references of the literature), whereas flavonoids were tested at a dosage established depending on their presence in the active extract. Treatments were evaluated 30 min (reference and flavonoids) and 60 min (extracts) after administration.

#### 2.5.1. PTZ-Induced Seizures

PTZ (80 mg/kg, i.p.) was used to induce clonic-tonic convulsions [20–22]. Then, latency to the onset of myoclonus, clonic-tonic, and tonic seizures was recorded; if no convulsion and mortality occurred within 30 min, the animals were considered to be protected from seizures. Diazepam (1 mg/kg, i.p.) was used as reference drug administered 30 min before seizures, whereas PTZ was injected 60 min after 100, 300, or 600 mg/kg, i.p. of the hexane, methanol, or aqueous inflorescences extracts.

Given that only methanol and aqueous extracts showed significant activity, it was interesting to analyze the possible anticonvulsant effect of these corresponding leave extracts at a dosage of 600 mg/kg. In the case of identified flavonoids, given that synergism among the constituents might be involved in the effect of the crude extract, it was decided to test the individual flavonoids in dosage approximate 10-fold more than that found in the crude extract. Control animals received the vehicle by the same route and volume of administration.

#### 2.5.2. High Performance Liquid Chromatography (HPLC) Analysis

**Figure 1:**
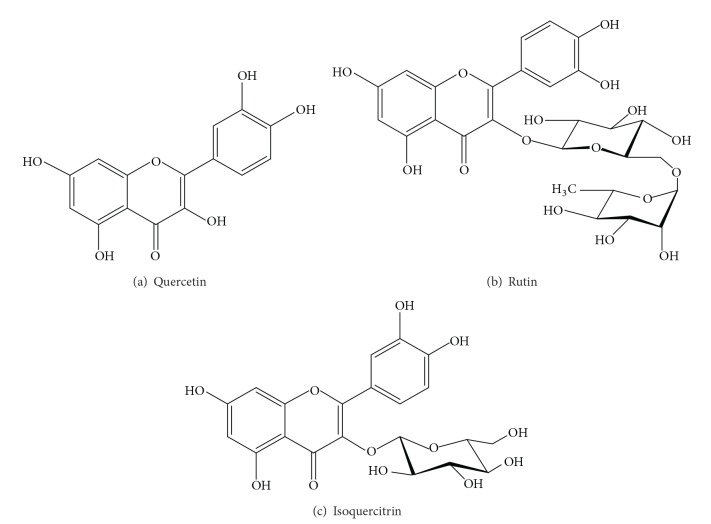
Flavonoid chemical structures.

A sample (1 mg) of each methanol extract was dissolved in 1 mL of methanol and filtered to inject 20 *μ*L to the HPLC system using an Agilent Technologies Chromatograph, ODS hypersil C-18 column (125 mm × 4 mm i.d., 5 *μ*m particle size), and UV detector (Agilent, G1365BMWD, multiwavelength detector). The system was run with an isocratic gradient of acetonitrile-water 15 : 85; water was acidulated with trifluoroacetic acid to reach 2.5 pH. The column was maintained at 30°C with a flow of 1 mL/min. Total running time was 14 min. The calibration curve was based on standards of previously reported flavonoids of* Tilia* species such as quercetin, rutin, and isoquercitrin (Figure 1). Chromatograms were detected at 350 nm.

### 2.6. *In Vitro* Antioxidant Activity of* T. americana* var.* mexicana* Extracts

#### 2.6.1. Radical Anion Superoxide (O_2_^∙−^) Scavenging Capacity

The xanthine oxidase system was used to determine the O_2_^∙−^ scavenging capacity of the* Tilia* extracts. Levels of O_2_^∙−^ in the assay system and xanthine oxidase activity were measured by an NBT reduction using a DU-640 series Beckman spectrophotometer. This system can be used to test the O_2_^∙−^ scavenging capacity only when the samples do not interfere with xanthine oxidase activity. A compound with O_2_^∙−^ scavenging capacity should decrease the NBT reduction without interfering with the xanthine oxidase activity measured as uric acid production. A total of 800 *μ*L of the following reaction mixture was mixed with 100 *μ*L of different concentrations of* Tilia* extracts: 90 *μ*M xanthine, 16 mM Na_2_CO_3_, 22.8 *μ*M NBT, and 18 mM phosphate buffer (pH 7.0). The reaction was started by the addition of 100 *μ*L of xanthine oxidase (168 U/L). The optical density was recorded at 295 nm for uric acid production and 560 nm for O_2_^∙−^ in the assay system [15]. The scavenging percentage was obtained from the optical density at 560 nm. NDGA (0, 1, 2, 3.02, and 8 *μ*g/mL) was used as a standard for O_2_^∙−^ scavenging in this assay.

#### 2.6.2. Hydroxyl (HO^∙^) Scavenging Capacity

The ability of* Tilia* extracts to scavenge HO^∙^ was assessed using the Fe^3+^-EDTA-H_2_O_2_-deoxyribose system [23]. The reaction mixture, containing deoxyribose (0.056 mM), H_2_O_2_ (1 mM), potassium phosphate buffer (10 mM, pH 7.4), FeCl_3_ (0.2 mM), EDTA (0.2 mM), and ascorbic acid (0.2 mM), was incubated in a water bath at 37 ± 0.5° C for 1 h. The extent of deoxyribose degradation caused by the formation of HO^∙^ was measured directly in the aqueous* Tilia* extract phase using the thiobarbituric acid test at 532 nm. The ability of these to scavenge HO^∙^ was compared with that of DMTU (0, 1, 2, 10, 20, and 31 *μ*g/mL).

#### 2.6.3. Hypochlorous Acid (HOCl) Scavenging Capacity

The catalase assay involved the spectral analysis of the enzyme. We generated samples for spectra of catalase (200–500 nm), catalase treated with HOCl, and catalase containing varied mixtures of HOCl treated with increasing concentrations of* Tilia* extracts and a reference compound. The HOCl scavenging capacity of these extracts or the reference antioxidant was evident by the inability of HOCl to eliminate/decrease in a concentration-dependent manner. Experiments were carried out essentially as described previously [24].

A solution of 49.8 *μ*M bovine liver catalase (16.6 *μ*M, final concentration) was mixed with 18 mM HOCl (6 mM, final concentration) in the presence of increasing concentrations of* Tilia* extracts or the reference compound of ascorbic acid (0, 22, 44, 88, and 176 *μ*g/mL). Spectra (370–450 nm) of catalase alone, catalase plus HOCl, or catalase plus HOCl and the* Tilia* extracts or the reference compound were generated, and the optical density (OD) at 404 nm was recorded. The OD of catalase alone minus the OD of catalase plus HOCl was considered to be 100% of catalase degradation (or 0% of scavenging activity).

The difference in the OD of the catalase alone minus the OD of the catalase plus HOCl in presence of either extracts or the reference compound was compared with this value and the ability of these to scavenge HOCl was compared with that of ascorbic acid.

#### 2.6.4. Determination of Hydrogen Peroxide (H_2_O_2_) Scavenging Capacity

A solution of 75 *μ*M H_2_O_2_ was mixed (1 : 1 v/v) with water (0% scavenging tube) or with different concentrations of* Tilia* extracts and incubated for 30 min at room temperature (22°C). Afterwards, H_2_O_2_ levels were measured. Briefly, 9 volumes of 4.4 mM BHT in HPLC-grade methanol were mixed with 1 volume of 1 mM xylenol orange and 2.56 mM ammonium ferrous sulfate in 0.25 M H_2_SO_4_ to give the working FOX reagent. Volumes of 45 *μ*L of each extract solution and 45 *μ*L of 75 *μ*M H_2_O_2,_ were dispensed in 1.5 mL Eppendorf tubes and mixed with 10 *μ*L of HPLC-grade methanol immediately followed by the addition of 0.9 mL of FOX reagent. Samples were then vortexed for 5 s and incubated at room temperature for 10 min. The tubes were centrifuged at 15,000 ×g for 10 min, and the absorbance at 560 nm was read against a methanol blank. The concentration of H_2_O_2_ was calculated from a standard curve prepared with increasing H_2_O_2_ concentrations. Sodium pyruvate (0, 22, 55, 110, 220, 550, and 1100 *μ*g/mL) was used as a standard for H_2_O_2_ scavenging activity [24].

#### 2.6.5. Determination of Singlet Oxygen (^1^O_2_) Scavenging Capacity

The production of ^1^O_2_ by NaOCl and H_2_O_2_ was determined using DMNA as a selective acceptor of ^1^O_2_ using a previously published method with minor modifications [24]. The bleaching of DMNA was monitored spectrophotometrically at 440 nm. The assay mixture contained 45 mM Na-phosphate buffer (pH 7.1), 10 mM histidine, 10 mM NaOCl, 10 mM H_2_O_2_, 50 *μ*M DMNA, and 0.1 mL of the* Tilia* extract. The total reaction volume (2.0 mL) was incubated at 30°C for 40 min. The extent of ^1^O_2_ production was measured by the decrease in the absorbance of DMNA at 440 nm. The relative scavenging efficiency (% inhibition production of ^1^O_2_) of the* Tilia* extracts was estimated using the difference in absorbance of DMNA with and without the addition of* Tilia* extracts and the reference compound. Glutathione (0, 0.92, 1.53, 2.15, 2.45, and 3.07 mg/mL) was used as a standard for ^1^O_2_ scavenging.

### 2.7. Statistical Analysis

All data are expressed as the mean ± SD for* in vitro* studies and as the mean ± SEM for* in vivo* studies. For* in vitro* studies, data were compared against the blank tube without* Tilia* extracts or the reference compounds using a one-way analysis of variance (ANOVA) followed by Dunnett's test (GraphPad Prism 4.0 Software, San Diego, CA, USA). A value of *P* < 0.05 was considered statistically significant. The scavenging capacity was expressed as the 50% inhibitory concentration (IC_50_) value, which denotes the concentration of* Tilia* extracts or the reference compounds needed to produce a 50% reduction in the oxidizing effect relative to the blank tube. For* in vivo* studies, statistical differences were analyzed using ANOVA followed by Tukey's test. A value of *P* < 0.05 was considered significant.

## 3. Results

### 3.1. The Anticonvulsants Effects on PTZ-Induced Seizures

**Figure 2:**
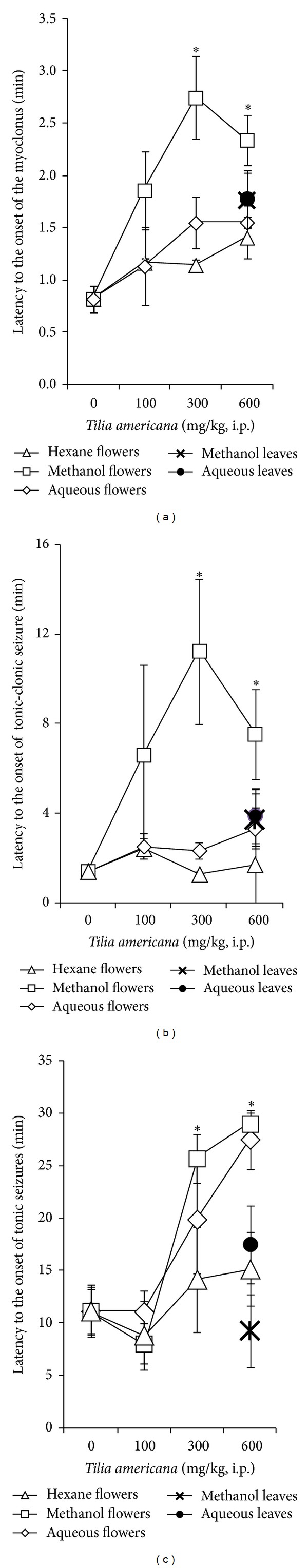
Effects of the organic and aqueous crude extracts from the* Tilia americana* var.* mexicana* inflorescences and leaves in the PTZ-induced myoclonus (a), tonic-clonic (b), and tonic seizures in mice. Each point represents the mean ± SE of at least 6 repetitions. *P < 0.05, ANOVA followed by Tukey's test.

PTZ (80 mg/kg) induced myoclonic (0.82 ± 0.12 min), clonic (1.41 ± 0.27 min), and tonic (13.10 ± 2.28 min) convulsions in 100% of control mice (Figures 2(a)–2(c)). In the* Tilia* inflorescences, the hexane extract did not significantly modify the onset of any convulsive behaviors (Figures 2(a)–2(c)).

Pretreatment with the methanol extract at 300 and 600 mg/kg delayed the onset of the first myoclonic (2.74 ± 0.46 min and 2.34 ± 0.21 min), generalized (clonic-tonic, 11.25 ± 3.48 min and 7.55 ± 2.15 min, *P* < 0.05), and tonic (25.68 ± 2.31 and 28.99 ± 1.01 min, *P* < 0.05) seizures induced by PTZ in mice (Figures 2(a)–2(c)). The aqueous extract of inflorescences significantly delayed only the presence of tonic seizures at doses of 300 mg/kg (19.89 ± 4.54 min) and 600 mg/kg (27.51 ± 2.49 min) (Figure 2(c)). Regarding leaves of* Tilia*, the methanol and aqueous extracts did not modify the onset of seizures at the highest dosage tested (600 mg/kg, i.p.) (Figures 2(a)–2(c)).

The percentage of animals showing myoclonic, clonic-tonic, and tonus behavior in the control group was 100%. By other side, a decreased in the percentage of mice showing tonic seizures was observed in the three tested inflorescence extracts as follows: (a) in hexane extract at 300 mg/kg (33%) and 600 mg/kg (16%); (b) in methanol extract at 300 mg/kg (33%) and 600 mg/kg (83%); (c) in aqueous extract at 100 mg/kg (16%), 300 mg/kg (50%); and 600 mg/kg (83%). Regarding the leaves extract, a reduction was observed in the presence of tonic seizures after methanol (16%) and aqueous (33%) extracts.

In reference to the flavonoids, the aglycone quercetin (100 mg/kg, i.p.) did not modify neither latency nor percentage of seizures in mice, whereas its glucosides rutin (quercetin-3-O-rutinoside) and isoquercitrin (quercetin-3-O-*β*-D-glucopyranoside) significantly delayed the presence of the first tonic seizure and reduced the percentage of mice showing this kind of convulsion (Table 1).

### 3.2. HPLC Analysis from Flowers and Leafs of* Tilia americana* var.* mexicana*

**Figure 3:**
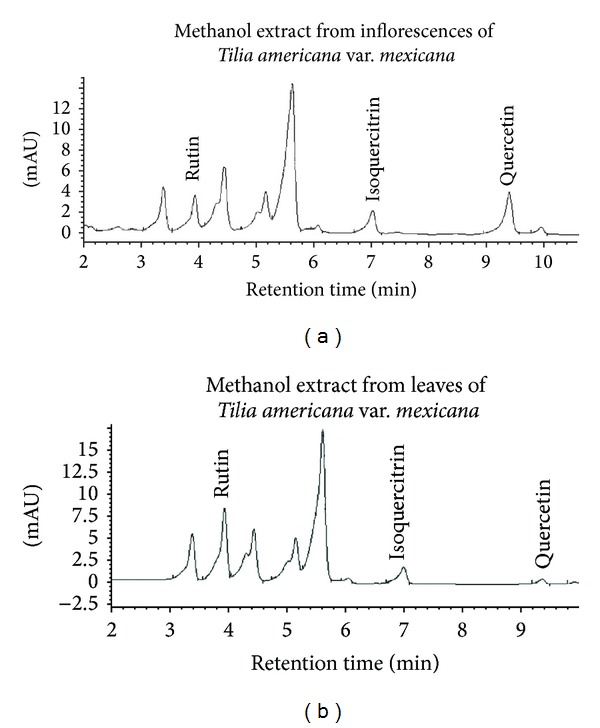
HPLC chromatogram demonstrating the flavonoids pattern of a methanol extract obtained from inflorescences (a) and leaves (b) of* Tilia americana* var.* mexicana*.

According to the concentration of flavonoids determined from three peaks in the HPLC analysis (Figure 3, Table 2), the presence of quercetin (aglycone), rutin, and isoquercitrin glycosides expected in the highest dosage (600 mg/kg) of methanol extracts from inflorescences and leaves administered in mice would be as follows: regarding inflorescences 12 mg of quercetin (Figure 3(a)), 2.11 mg of rutin (Figure 3(b)), and 1.04 mg of isoquercitrin (Figure 3(c)), but in the leaves 0.54 mg of quercetin, 5.36 mg of rutin, and 0.74 mg of isoquercitrin.

### 3.3. Antioxidant Effects on Free Radical Scavenging

All the* Tilia* extracts successfully scavenged the reactive species studied. The analysis of the IC_50_ values indicated that both tested methanol and hexane extracts of* Tilia* (leaves and inflorescences) display scavenging activity in a concentration-dependent manner.

From* Tilia* inflorescences, methanol extract was less efficient than NDGA, ascorbic acid, pyruvate, and DMTU (*P* < 0.05) but 1.0-fold more efficient than GSH in scavenging ^1^O_2_ (Table 3), whereas hexane extract was less efficient than NDGA, ascorbic acid, pyruvate, and DMTU (*P* < 0.05), but 2.0-fold more efficient than GSH in scavenging ^1^O_2_ (Table 3). In the case of the aqueous extract, it was less efficient than NDGA, ascorbic acid, pyruvate, GSH, and DMTU (*P* < 0.05) (Table 4). Regarding* Tilia* leaves, methanol was less efficient than NDGA, ascorbic acid, pyruvate, and DMTU (*P* < 0.05) but 9.5-fold more efficient than GSH in scavenging ^1^O_2_ (*P* < 0.05) (Table 4). Hexane extract from leaves was less efficient than NDGA, ascorbic acid, pyruvate, GSH, and DMTU (*P* < 0.05) (Table 4). Finally, aqueous extract from leaves was unable to scavenge the studied reactive species (data not shown). In accord with the obtained results, the efficiency in scavenging ^1^O_2_ was in the following order: leaves methanol extract > inflorescences hexane extract > inflorescences methanol extract.

## 4. Discussion

In this study, inflorescences and leaves of* T. americana,* prepared as organic and aqueous extracts, were investigated by analyzing its anticonvulsant and antioxidant properties, as well as the differences in some flavonoids concentrations. Significant differences were found in both activities among the analyzed extracts and flavonoids constituents that depended on the inflorescences or leaves studied. It is the first evidence of the anticonvulsant properties of the inflorescences and leaves of* Tilia*. In particular, methanol and aqueous extracts were effective against tonic seizure, but methanol extract was more active in generalized seizure or myoclonus; these results suggest more concentrated presence of flavonoids such as quercetin implicate major anticonvulsant response. Nevertheless, the individual evaluation of the flavonoids quercetin, isoquercitrin, and rutin demonstrated that among them exists differential capability to inhibit seizures.

It is known that repeated or single-dose administration of PTZ decreases GABAergic function producing seizures [25]; it is due to its selective blocker action on the chloride ionosphere complex to the *γ*-aminobutyric acid_A_ (GABA_A_) receptor. It is also known that oxidative stress in the CNS has been shown in several experimental models of epilepsy, such as the PTZ kindling model [26–28].

Oxidative stress is defined as an imbalance in the levels of reactive oxygen species (ROS). Generation of ROS is ubiquitous because they are generated during aerobic metabolism, that is, mitochondrial oxidation and other monoamine oxidants. Various defense systems exist in the brain to scavenge ROS, including glutathione peroxidase, vitamin E, vitamin C, and vitamin A [27, 29]. In a previous study, pretreatment with hexane or aqueous extracts of the* Tilia* inflorescences prevented dysfunctional response of the ischemic tissue in a significant manner suggesting a neuroprotector role [30]. The reduced response in ischemic tissues can be prevented by pretreatment with certain agents, such as antioxidants or calcium channels blockers, like verapamil, considered as neuroprotective drugs [31, 32]. In this study, we demonstrated that not only the methanol but also a hexane extract from the leaves and flowers contains free radical scavengers that are more concentrated in the methanol extract of the leaves against ^1^O_2_.* Tilia* extracts also display an improved scavenging capacity when compared with the reference compounds suggesting that these possess a neuroprotective activity that might be involved against PTZ-induced seizures due to its antioxidant activity.

Antioxidant and anticonvulsive abilities of natural products have already been demonstrated. In 2010, Aldarmaa et al. [33] showed that ethanol extract of the aerial parts and roots from* Astragalus mongholicus* trapped hydroxyl radicals (IC_50_ root = 4.7 mg/mL; IC_50_ aerial parts = 7.8 mg/mL; reference compound = 6.6 mg/mL), diminished lipid peroxidation (IC_50_ root = 2.04 mg/mL; IC_50_ reference compound = 7.92 mg/mL), and protected against malondialdehyde-induced oxidative damage by ameliorating effects on mitochondrial complexes I and II, malate dehydrogenase, and the mitochondria membrane potential (concentrations from 18 to 72 mg/mL). Moreover, an* Astragalus mongholicus* extract (150 and 300 mg/kg) diminished malondialdehyde, protein carbonyl, and ROS levels induced by PTZ in mice [33]. The* Astragalus mongholicus* extract significantly increased the latency of PTZ-induced seizures and decreased the duration of seizures at doses of 150 and 300 mg/kg [33]. In 2009, Ilhan et al. showed that* Nigella sativa* oil (12 mL/kg) significantly decreased lipid peroxidation and increased SOD and GPx activity in the brain tissue of PTZ-treated group being more effective at reducing the seizures than valproate (100 mg/kg) [26].

*Tilia* extracts possess capacity to trap multiple reactive species, including O_2_^∙−^, HO^∙^, HOCl, H_2_O_2,_ and ^1^O_2_. These results suggest that free radial scavenging might be a part of the mechanisms underlying the antinociceptive, anxiolytic, and sedative effects previously observed in experimental models and in human use of traditional medicine for* T. americana* [7, 9, 16, 18, 34]. The methanol extract of leaves showed a significantly higher scavenger activity against ^1^O_2_ in comparison with the reference compound GSH. It has been demonstrated that antioxidant properties of plants depend on the contain of constituents in leaves, inflorescences, and fruits as well as chemical profile, extract type, part of the plant, species, light exposition, life cycle, and time of plant germination, flowering, and harvest [35–37]. Phytochemical studies have reported that flavonoids, such as quercitrin, isoquercitrin, kaempferol, astragalin, hyperoside, tiliroside, quercetin-3,7O-dirhamnoside, and kaempferol-3,7-*O*-dirhamnoside, are major components of methanol extracts from the* Tilia* genus [8, 18]. The presence of flavonoids in the methanol extract suggests that the anticonvulsant properties shown by* Tilia* could be due to these compounds, which in previous studies have been involved as responsible anticonvulsants and anxiolytics [16, 38]. This is in agreement with the recent study of our group demonstrating that ethyl acetate extract of* T. americana* var.* mexicana* possesses high concentration of flavonoids and potential scavenging against ^1^O_2_ [39].

Based on our present findings, we suggest that the anticonvulsant effects of polar extracts of* Tilia* may be mediated by reducing oxidative damage in addition to GABAergic and serotoninergic neurotransmission as previously reported. Furthermore, it is important to consider* Tilia* as an antioxidant that may be a possible therapeutic agent for other neuropathies related to oxidative stress, such as Parkinson's disease [40–44] and Alzheimer's disease [32, 45–47].

*Tilia* species have been used in traditional medicine for their tranquilizing properties by using exclusively the inflorescences (flowers and bracts). It has been observed that during the flowering months (April to June) there is an increase in the marketing of inflorescences of this species, given the belief that the medicinal effect is greater when the infusion of inflorescences is prepared during this period [2]. Our results corroborated that inflorescences of* Tilia* produce inhibitory activity in the CNS as an anticonvulsive as reported by people using folk medicine. Nevertheless, it is important to mention that differences in the anticonvulsant activity were encountered when* Tilia* leaves were tested in comparison to the inflorescences. These findings reinforce the medicinal properties of this plant and provide evidence that leaves are not as recommended for this medicinal purpose as it is for inflorescences. The results demonstrated that the presence of flavonoids differs depending on the part of the plant assayed. Major concentrations were observed in the case of quercetin and rutin; however isoquercitrin was more active than these two flavonoids. It has been demonstrated that flavonoids like quercetin, rutin, and kaempferol elicit CNS activity which involves the inhibitory GABAergic system [5, 48], but it is the first time to our knowledge that CNS activity is described for isoquercitrin.

The precise mechanisms of action of flavonoids might differ depending on the kind of chemical structure and pharmacological activity, but regarding the antianxiety and sedative-like activities the involvement of the GABAergic and serotonergic systems is the most reported [49–51]. In our study, an antioxidant activity was demonstrated for the hexane, methanol, and aqueous crude extracts from inflorescences and leaves and some flavonoids (quercetin, rutin, and isoquercitrin) of* Tilia*. From these flavonoids, only rutin and isoquercitrin (glycosides of quercetin) were observed to be partial responsible for the anticonvulsant activity of this species suggesting that a synergism among these and likely other constituents are involved in their anticonvulsant activities whose antioxidant properties might also be participating in their neuroprotective effects.

It is recognized that glycosidic moiety increases hydrophilicity influencing pharmacokinetic properties in the body fluids. Glycosylation can strongly influence transport of flavonoids through haematoencephalic barrier modifying the entrance into the brain tissue and its neuropharmacological properties [52]. In particular, the high level of quercetin confers to the extracts an important antioxidant potential, while phenolic compounds are very important plant constituents due to their scavenging abilities associated with their OH groups and to their capacity to chelate transition metal ions [53].

The literature data corroborate the structural features of flavonoids including (i) the ortho dihydroxy structure in the B-ring; (ii) the C2–C3 double bond conjugated with the 4-oxo group; and (iii) the presence of hydroxyl groups in positions 3 and 5. These characteristics are important for the flavonoids free radical scavenging activities [54]. The predominant mechanism of action of catechols is probably via donation of a single electron to the radical cation resulting in the formation of a semiquinone, which can donate a further electron to form the quinine [55].

On the other hand, it has been demonstrated that quercetin and kaempferol flavonoid aglycones isolated from methanol-water extract from* T. platyphyllos* are stronger scavengers of ^1^O_2_ in comparison with Trolox (vitamin E hydrophilic derivative) [56]. It has been demonstrated that HO groups located on the B-ring in flavonoids enhance reactivity against O_2_^∙−^, ABTS, and ^1^O_2_. [57]. Quercetin with a catechol structure on B-ring (two HO groups in positions 3′ and 4′) showed 4-time higher reactivity towards ^1^O_2_ in comparison with kaempferol (one HO group in position 4′) [57]. Additionally, the relevance of hydroxyl group activating the double bond of the C-ring in flavonols in ^1^O_2_ quenching has been established. In general, flavonol glycosylated forms are weaker antioxidants than their respective aglycones [57]. We would expect more antioxidant activity with quercetin in comparison with isoquercitrin and rutin.

Finally, our results demonstrated the anticonvulsant and antioxidant activities in the inflorescences and leaves of* Tilia* species that involve the presence of flavonoids. The differences in the pattern and concentrations of these compounds were factors that modify the anticonvulsant properties of* Tilia*, but quercetin may be useful for the standardization of the extract.

## 5. Conclusion

In summary, our results showed that polar extracts of* T. americana* var.* mexicana* significantly prevented severity of PTZ-induced seizures and attenuated oxidative stress levels involving the presence of flavonoids such as quercetin, rutin, and isoquercitrin. These extracts and their constituents are part of the anticonvulsant activity that are described in folk medicine.

## Figures and Tables

**Table 1:** Anticonvulsive effects of some flavonoids in the pentylenetetrazole-induced seizures in mice.

Treatment	Dose (mg/kg, i.p.)	Latency to the onset (min) and percentage of mice showing seizure					
		Myoclonus		Tonic-clonic		Tonic	
Vehicle	0	0.82 ± 0.12	100%	1.41 ± 0.27	100%	11.14 ± 1.15	100%
Quercetin	100	1.22 ± 0.23	100%	1.40 ± 0.24	100%	12.12 ± 2.21	100%
Rutin	30	1.25 ± 0.21	100%	2.46 ± 0.82	100%	21.83 ± 4.80∗	33%
Isoquercitrin	10	0.80 ± 0.07	100%	1.04 ± 0.11	100%	23.87 ± 4.11∗	33%

Data are represented as the mean ± SE of three independent assays. ∗*P* < 0.05, ANOVA followed by Tukey's test.

**Table 2:** HPLC analysis of the flavonoid pattern in methanol extract of samples of *Tilia americana *var. *mexicana *inflorescences and leaves collected in Puebla, Mexico.

Part of the plant	Flavonoid	concentration	(*μ*g/mg)
	Quercetin	Rutin	Isoquercitrin
Inflorescences	20.09 ± 1.20	3.52 ± 0.21	1.74 ± 0.01
Leaves	3.39 ± 0.10	8.94 ± 0.45	1.24 ± 0.13

Data are expressed as the mean ± SE of three samples.

**Table 3:** Scavenging capacity of the hexane, methanol, and aqueous extracts from *Tilia* inflorescences and references compounds.

Species	Hexane	Methanol	Aqueous		Reference compound
	IC_50_ (*μ*g/mL)	IC_50_ (*μ*g/mL)	IC_50_ (*μ*g/mL)		IC_50_ (*μ*g/mL)
O_2_^•−^	70.21 ± 1.21∗	51.11 ± 1.82∗	421.82 ± 7.85∗	NDGA	1.027 ± 0.165
HOCl	92.97 ± 3.43∗	173.46 ± 2.59∗	5764.83 ± 1341.1∗	Ascorbic acid	0.50 ±0.005
H_2_O_2_	34.48 ± 1.60∗	59.41 ± 2.57∗	1926.8 ± 1284.77∗	Pyruvate	0.120 ± 0.094
^ 1^O_2_	3.75 ± 1.10∗	7.46 ± 1.28∗	10897.1 ± 1171.21∗	GSH	7.58 ± 0.25
HO^•^	>146.8	45.13 ± 1.94∗	>10130	DMTU	1.22 ± 0.01

Data are represented as the mean ± SE of three independent assays. ANOVA followed by Dunnett's test. ∗*P* < 0.05 versus reference compounds.

**Table 4:** Scavenging capacity of the hexane and methanol extracts of *Tilia* leaves and reference compounds.

Species	Hexane	Methanol		Reference compound
	IC_50_ (*μ*g/mL)	IC_50_ (*μ*g/mL)		IC_50_ (*μ*g/mL)
O_2_^•−^	57.32 ± 0.58∗	27.64 ± 1.61∗	NDGA	1.027 ± 0.165
HOCl	16.67 ± 1.89∗	225.26 ± 5.30∗	Ascorbic acid	0.50 ± 0.005
H_2_O_2_	97.96 ± 4.10∗	73.25 ± 3.02∗	Pyruvate	0.120 ± 0.094
^ 1^O_2_	23.28 ± 1.15∗	0.80 ± 0.001∗	GSH	7.58 ± 0.25
HO^•^	172.4 ± 2.05^+^	60.12 ± 1.98∗	DMTU	1.22 ± 0.01

Data are represented as the mean ± SE of three independent assays. ANOVA followed by Dunnett's test. ∗*P* < 0.05 versus reference compounds.
